# Resource-rich Intensive Care Units Improve Health―What Is Next?

**DOI:** 10.31662/jmaj.2021-0229

**Published:** 2022-03-25

**Authors:** Nobuaki Shime

**Affiliations:** 1 Department of Emergency and Critical Care Medicine, Graduate School of Biomedical & Health Sciences, Hiroshima University, Hiroshima, Japan

**Keywords:** intensive care unit, mortality, intensivist

## To the Editor:

I recently read the study by Ohbe et al.^(1)^ published in the latest issue of the *Japan Medical Association Journal* with great interest. In their study, Ohbe et al. investigated the association between resource-rich intensive care units (ICUs) and patient mortality using large-scale Japanese inpatient database (Diagnosis Procedure Combination inpatient database). They found that resource-rich ICUs were associated with lower ICU mortality. The authors should be admired for elucidating the importance of structural factors in outcomes in a critically ill population wherein outcome-improving interventions are quite limited.

The results, however, should be read with some caution, as suggested by the authors in their discussion of the study limitations. Whether resource richness in ICU itself had a direct impact on outcomes remains unclear. Rich ICUs are probably located in larger, academic hospitals with a higher number of ICU beds. A previous study published by the same group has suggested a volume-outcome relationship specifically in hospitals with a higher ICU-to-hospital bed ratio^(2)^. The factors that could affect the outcome, including the dependency on intensivists^(3)^, multidisciplinary care with multiple physicians and healthcare providers, patient volumes, or all of these, should be further elucidated.

Where should the hospital administrators or the policymakers focus next? It is probably based on the findings in this study that resource-rich ICUs improve health; however, this raises the question of how we could achieve “common prosperity.” Should resource-rich ICUs that provide better health outcomes become prevalent throughout the country? Alternatively, should critically ill patients be centralized in more resource-rich ICUs? In fact, the proportion of resource-rich ICUs increased from 7% in 2014 to 34% in 2019, and the number of board-certified intensivists has doubled since 2014 [Data from the Japanese Society of Intensive Care Medicine]. The provision of additional evidence to guide policymakers in modifying ICU system design remains necessary in Japan.

*“To get rich is glorious.” -Deng Xiaoping*

## Article Information

### Conflicts of Interest

None

### Acknowledgement

We thank John Holmes, MSc, from Edanz (https://jp.edanz.com/ac) for editing a draft of this manuscript.

### Author Contributions

The author solely contributed all of the work for the manuscript.
